# Growth Decline Linked to Warming-Induced Water Limitation in Hemi-Boreal Forests

**DOI:** 10.1371/journal.pone.0042619

**Published:** 2012-08-15

**Authors:** Xiuchen Wu, Hongyan Liu, Dali Guo, Oleg A. Anenkhonov, Natalya K. Badmaeva, Denis V. Sandanov

**Affiliations:** 1 Department of Ecology, College of Urban and Environmental Sciences, Peking University, Beijing, China; 2 Institute of General and Experimental Biology, Siberian Branch of the Russian Academy of Sciences, Ulan Ude, Russia; Ohio State University, United States of America

## Abstract

Hemi-boreal forests, which make up the transition from temperate deciduous forests to boreal forests in southern Siberia, have experienced significant warming without any accompanying increase in precipitation during the last 80 years. This climatic change could have a profound impact on tree growth and on the stability of forest ecosystems in this region, but at present evidence for these impacts is lacking. In this study, we report a recent dramatic decline in the growth of hemi-boreal forests, based on ring width measurements from three dominant tree-species (*Pinus sylvestris, Larix sibirica* and *Larix gmelinii*), sampled from eight sites in the region. We found that regional tree growth has become increasingly limited by low soil water content in the pre- and early-growing season (from October of the previous year to July of the current year) over the past 80 years. A warming-induced reduction in soil water content has also increased the climate sensitivity of these three tree species. Beginning in the mid-1980s, a clear decline in growth is evident for both the pine forests and the larch forests, although there are increasing trends in the proxy of soil water use efficiencies. Our findings are consistent with those from other parts of the world and provide valuable insights into the regional carbon cycle and vegetation dynamics, and should be useful for devising adaptive forest management strategies.

## Introduction

Boreal forests in the northern hemisphere are predicted to be a major sink for atmospheric CO_2_ as the global climate warms [1], [2]. Satellite observations, atmospheric CO_2_ measurements and the results of dynamic vegetation models have shown that climate warming leads to increased tree growth and a marked greening trend in boreal forests [3], [4], [5]. However, there are also reports of extensive tree growth decline or mortality at northern mid- and high- latitudes [6], [7], [8], [9], which highlights the large tempo-spatial variability in tree growth responses to climate change in boreal forests [10].

Given that tree growth is apparently declining rapidly in many parts of the world, even under relatively modest increases in global mean temperature and a drying climate [8], broad-scale climate-induced stress on tree growth could be expected to accompany projected future climate patterns [11]. In particular, warming-induced drought may trigger an extensive decline in the growth of hemi-boreal forests. A recent study showed that the decline in growth of *Larix siberica* in hemi-boreal forests is closely related to increased drought stress [9]. However, it is far from clear about the responses of tree growth to potential warming-induced drought.

**Figure 1:**
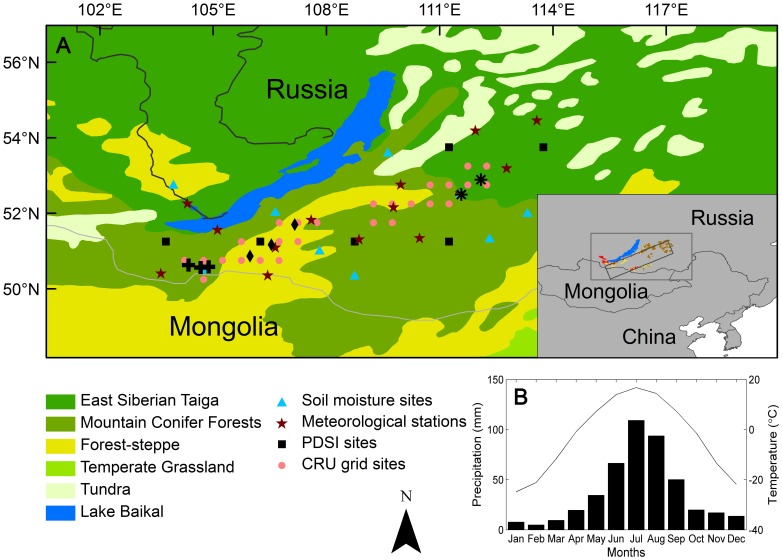
Geographical position (A) and climate diagram (B) of the study region. The sample sites of *Larix sibirica*, *Pinus sylvestris* and *Larix gmelinii* are indicated by black crosses, black diamonds and black asterisks, respectively. The approximate location of the selected transect (as indicated by the italic rectangle) and schematic distribution (according to the Forests of the USSR Map, 1990) of the three species (*L. sibirica*, *L. gmelinii* and *P. sylvestris*, as indicated by red, brown and yellow small patches, respectively) in and around the transect are shown in the inlet figure in (A). The mean monthly temperature (line) and total monthly precipitation (bars) during 1928–2006 are shown in (B) with data derived from dataset CRU TS 3.0 (http://www.cru.uea.ac.uk/).

Climate change models predict warmer temperatures in the hemi-boreal forests of southern Siberia [11], where there are three dominant species (*P. sylvestris, L. sibirica* and *L. gmelinii*). This warming may trigger a reduction in soil water content, thus further constraining tree growth and potentially leading to profoundly altered forest dynamics, such as large-scale tree die-off [8]. Previous studies have also found that the warming-induced decline in growth persists despite the fact that the water use efficiency of trees shows an increasing trend in many regions [12], [13], [14]. This background leads to two critical questions: (1) Has growth decline occurred in this region, and if so, what are the driving forces for this decline? (2) Do the responses of tree growth to climate differ between different species linked to the stand conditions? To answer these questions, we examined the relationship between tree growth and climate over the past 80 years in this region, based on a tree ring network (Fig. 1A). Answers to these questions will provide us with insights into the regional carbon cycle, vegetation dynamics and forest management in this and other hemi-boreal forests world-wide.

## Results

### Temporal Trends in Tree Growth and Common Variance

**Figure 2:**
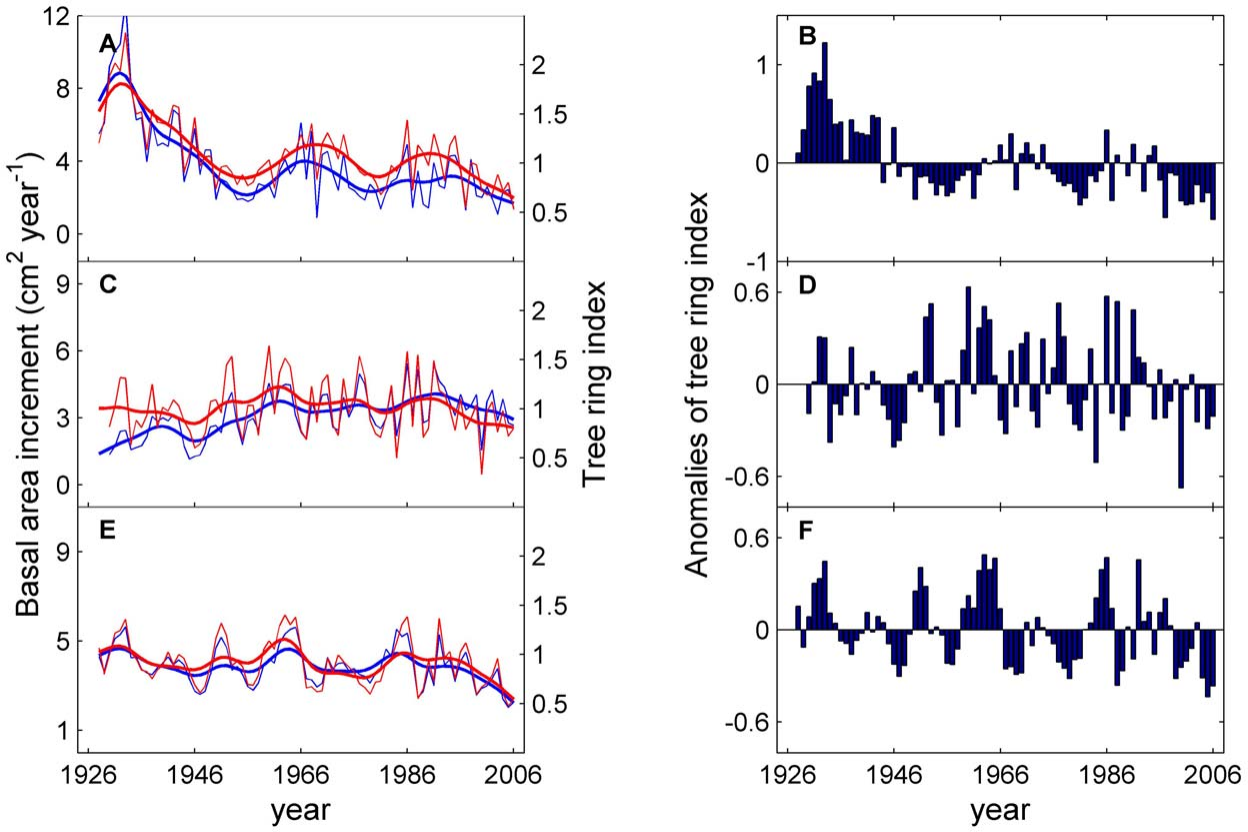
Basal area increments (blue lines), tree ring indices (red lines) and the anomalies of tree ring indices (bars) for *P. sylvestris* (A and B), *L. sibirica* (C and D) and *L. gmelinii* (E and F). Bold lines in the figure are the fitted cubic smoothing splines for basal area increments and tree ring indices.

**Figure 3:**
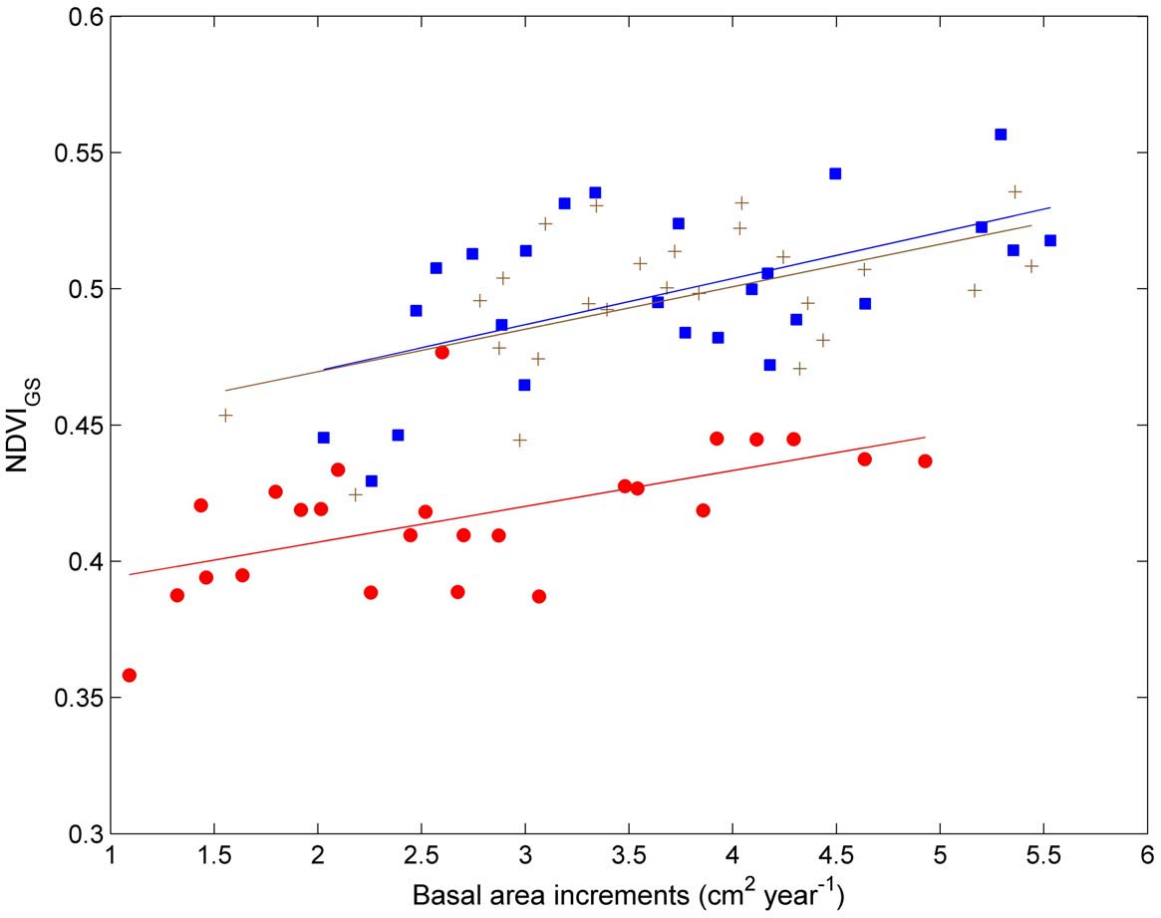
Basal area increments versus growing season average normalized difference vegetation index (NDVI) during 1982–2006 for *P. sylvestris* (red circle), *L. sibirica* (brown cross) and *L. gmelinii* (blue square). The lines are linear fits of the relationship between basal area increments and NDVI for *P. sylvestris* (*y* = 0.38+0.013*x*, *r* ^2^ = 0.33, *p*<0.001), *L. sibirica* (*y* = 0.44+0.016*x*, *r* ^2^ = 0.30, *p*<0.001) and *L. gmelinii* (*y* = 0.44+0.017*x*, *r* ^2^ = 0.32, *p*<0.01).

The BAI series consistently tracked the standardized TRI series for all three species between 1928 and 2006 (Figs. 2A, C, E). Trends in the BAI and TRI series show that *P. sylvestris* has undergone a remarkable decline in growth since 1928, despite the high variability highlighted by the smoothing fitted cubic spline (Figs. 2A). In contrast, the tree growth for larch species appears to have remained relatively stable (*L. gmelinii*), or even to have increased (*L. sibirica*), during these early periods (e.g. before the mid-1980s), and to have suffered a dramatic decline over the last 20 years of the record (Figs. 2C, E). The fitted linear regression shows the decline rates of BAI to be 0.70 (*p*<0.05) and 0.78 cm^2^ per decade (*p*<0.05) for *L. sibirica* and *L. gmelinii* for the last two decades (1986–2006) respectively. Overall, although temporal changes in growth patterns differ between these three species, all have experienced a decline in growth since approximately the middle of 1980s. Negative anomalies in the TRI series also appear to have been becoming increasingly prominent over the last two decades (Fig. 2B, D, F). The stand-level basal area increment measures of tree growth show close positive relationships to the growing season average NDVI for the period 1982–2006 for all the three species, as can be seen from the linear regression fits (Fig. 3). This result demonstrates that our sample data is representative of larger scale tree growth patterns in the region, and suggests that the hemi-boreal forests may be suffering a recent dramatic decline in growth, which has been especially severe since the late 1980s.

**Figure 4:**
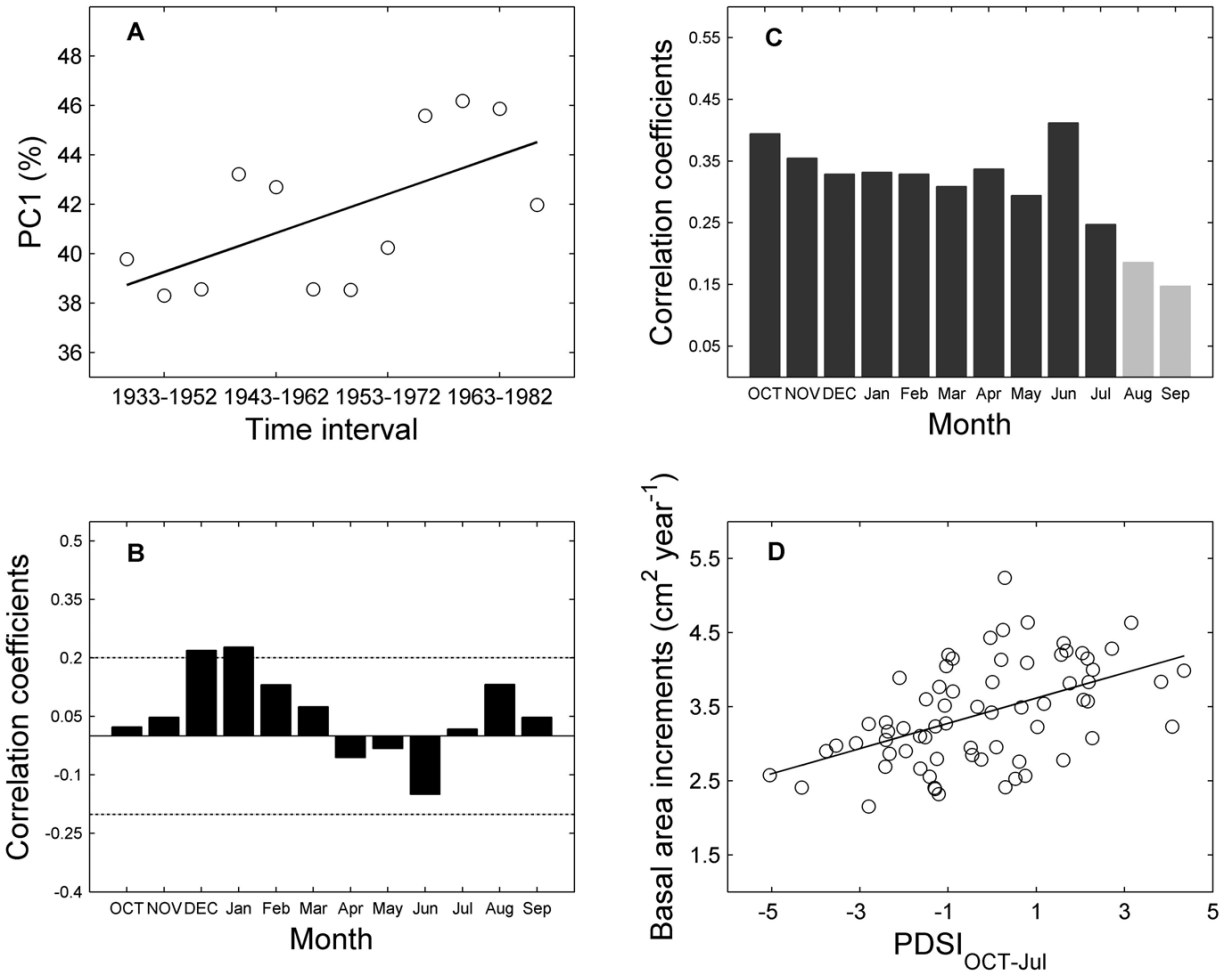
Evolution of shared growth variability explained by the first principal component (PC1) and relationships between PC1 and climate. Evolution of shared growth variability (estimated by the PC1) since 1928 using subintervals of 20 years with a 5-year lag was shown in (A) with a linear fit (black line, *y* = 0.53*x*+38.21, *r* ^2^ = 0.38, *p = *0.032). Simple correlations performed between the first principal component (PC1) and monthly temperature during 1928–2006 (B) and PDSI during 1937–2005 (C). The dotted lines in (B) indicate the 95% confidence intervals. Dark grey bars in (C) are statistically significant (*p*<0.05). The relationship between the regional BAI series and the average PDSI values through October of prior year to July of current year during 1937–2005 was shown in (D) with a linear fit (black line, *y* = 0.17*x*+3.45, *r* ^2^ = 0.25, *p*<0.001).

The first and second PCs (PC1 and PC2) of the chronology network PCA are significant, representing 42.1% and 18.7% of the total variance respectively. The scatter plot of the PCA loading coefficients reveals groups of chronologies with similar growth patterns (Fig. *S3*). Although the 8 chronologies are associated with different loadings for PC1, all 8 are positively correlated with it, showing that they share a common variance. *P. sylvestris* chronologies were mainly negatively correlated with PC2, while the two larch species chronologies generally had a positive correlation with PC2 (Fig. *S3*). The shared growth variability (PC1) for all the chronologies shows a marked increase (*r* ^2^ = 0.38, *p*<0.05) for recent decades (Fig. 4A).

### Regional and Species Growth-climate Relationships

Correlation analyses between the first principle component (PC1) and mean monthly temperature show that the monthly temperature significantly affects tree growth only in December of the previous year and January of current year for the study period (Fig. 4B). In contrast, all monthly PDSI values between October of the previous year and July of the current year are significantly related to regional tree-growth (Fig. 4C). The results of the step-wise linear regression between PC1 and monthly-, growing season-, pre-growing season (October of the previous year to April of the current year)- average temperature and PDSI, as well as PDSI for October to July (PDSI_OCT-Jul_), show that PDSI_OCT-Jul_ contributed most to the variability in regional tree growth between 1937 and2005 (*p*<0.05). The close relationship found between the regional BAI and PDSI_OCT-Jul_ confirms our results (Fig. 4D).

**Figure 5:**
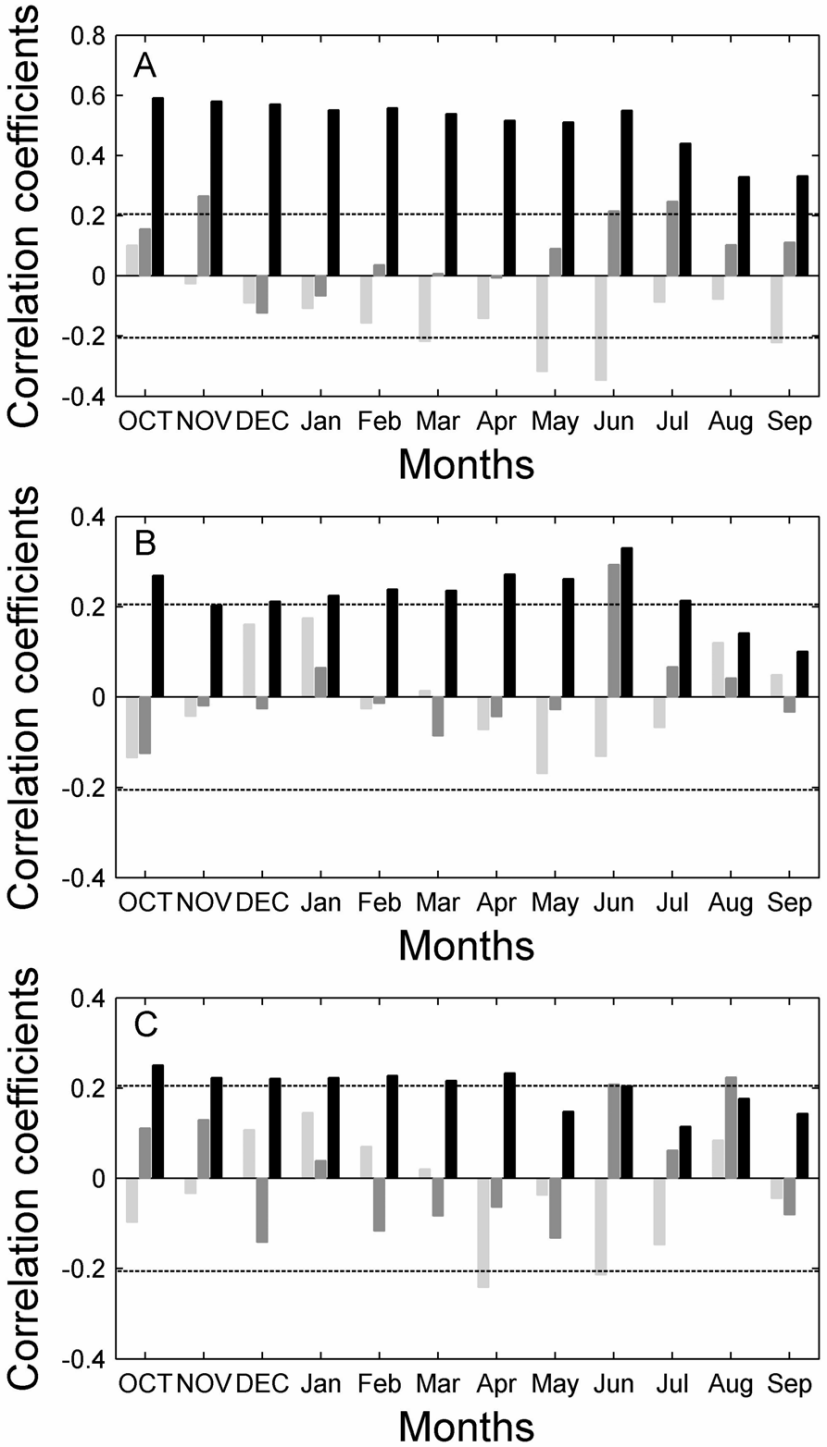
Correlations between the standard chronology and temperature (light grey bars), precipitation (dark grey bars), and PDSI (black bars) during 1937–2005 regarding *P. sylvestris* (A), *L. sibirica* (B) and *L. gmelinii* (C). The dotted lines indicate the 95% confidence intervals.

We further assessed the growth-climate relationships for the three species and identified some clear differences between the tree growth-climate responses of *P. sylvestris* and that of the two larch species. The tree growth of *P. sylvestris* is significantly limited (negative correlation) by the mean monthly temperature in the months during, and immediately prior to, the growing season, e.g. March, May, June and September (Fig. 5A). Conversely, larch tree-growth does not appear to be significantly affected by changes in temperature (*L. sibirica*), and seems to be limited by mean monthly temperature only during the pre- and early growing season, e.g. April and June (*L. gmelinii*) (Figs. 5B, C). Precipitation during both the growing season (e.g. June and July) and the pre-growing season (e.g. previous November) had a great effect on tree growth for *P. sylvestris* (Fig. 5A). In contrast, the larch growth is only correlated significantly to the precipitation for the growing season (Figs. 5B, C). *P. sylvestris* shows a significant and positive correlation to PDSI for all months, which differs from the results for larch, which show a significant and positive correlation to PDSI for the pre- and early growing season (Figs. 5A–C).

### Climate Sensitivity of Tree Growth

**Figure 6:**
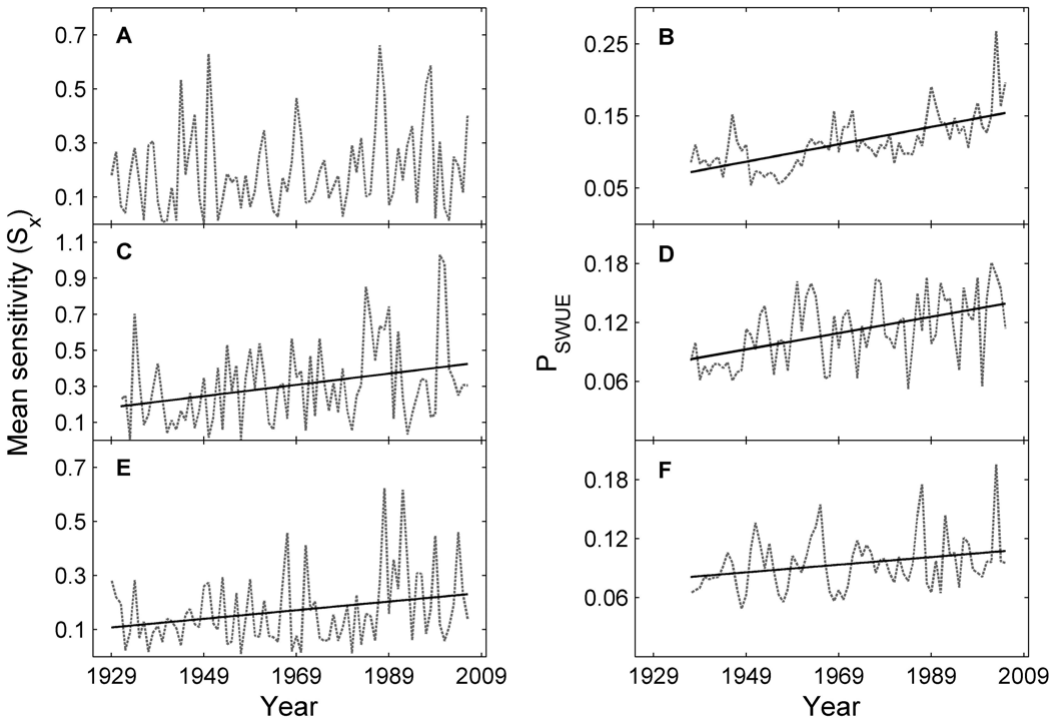
Temporal changes of mean sensitivity (*S_x_*, 1928–2006) and a proxy of soil water use efficiency (P_SWUE_, 1937–2005) relating to *P. sylvestris* (A, B), *L. sibirica* (C, D) and *L. gmelinii* (E, F). Lines in this figure are linear fits of *S_x_* and P_SWUE_ for *P. sylvestris* (*y* = 0.0012*x–*2.26, *p*<0.001), *L. sibirica* (*y* = 0.0027*x–*4.93, *p*<0.05, *y* = 0.00083*x–*1.53, *p*<0.001) and *L. gmelinii* (*y* = 0.0016*x–*2.97, *p*<0.05; *y* = 0.00033*x*–0.55, *p*<0.05) if there are significant trends as detected using Mann-Kendall test.

Despite high interannual variation, the mean sensitivity of *L. sibirica* and *L. gmelinii* increased markedly over the period 1928–2006, as illustrated by the results of both Mann-Kendall test (Z values are 2.44 and 2.24, respectively) and the linear regression fits (Figs. 6C, E). The P_SWUE_ of all three species increased dramatically from 1937 to 2005, as illustrated by the Mann-Kendall trend test (Z values are 5.86, 4.09 and 2.07 respectively, *p*<0.05) and by the linear regression fits (Figs. 6B, D, F).

### Climate Trends and Warming-induced Soil Water Limitation

Significant increases were seen in nearly all monthly temperatures during this period, as shown by the Mann-Kendall test. The estimated linear rate of temperature increase ranges from 0.13°C per decade in August to 0.51°C per decade in February, when considering data spanning the last 80 years (data are not shown here). The most dramatic increase in temperature was seen in the pre- and early growing season. No obvious trends were evident in the total monthly precipitation for the same period (except marked decreases in February and March, with linear slopes of −0.03 and −0.05 mm year^−1^ respectively), despite the high variability evident in the record. Notably, all monthly PDSI series have decreased markedly since 1937.

**Figure 7:**
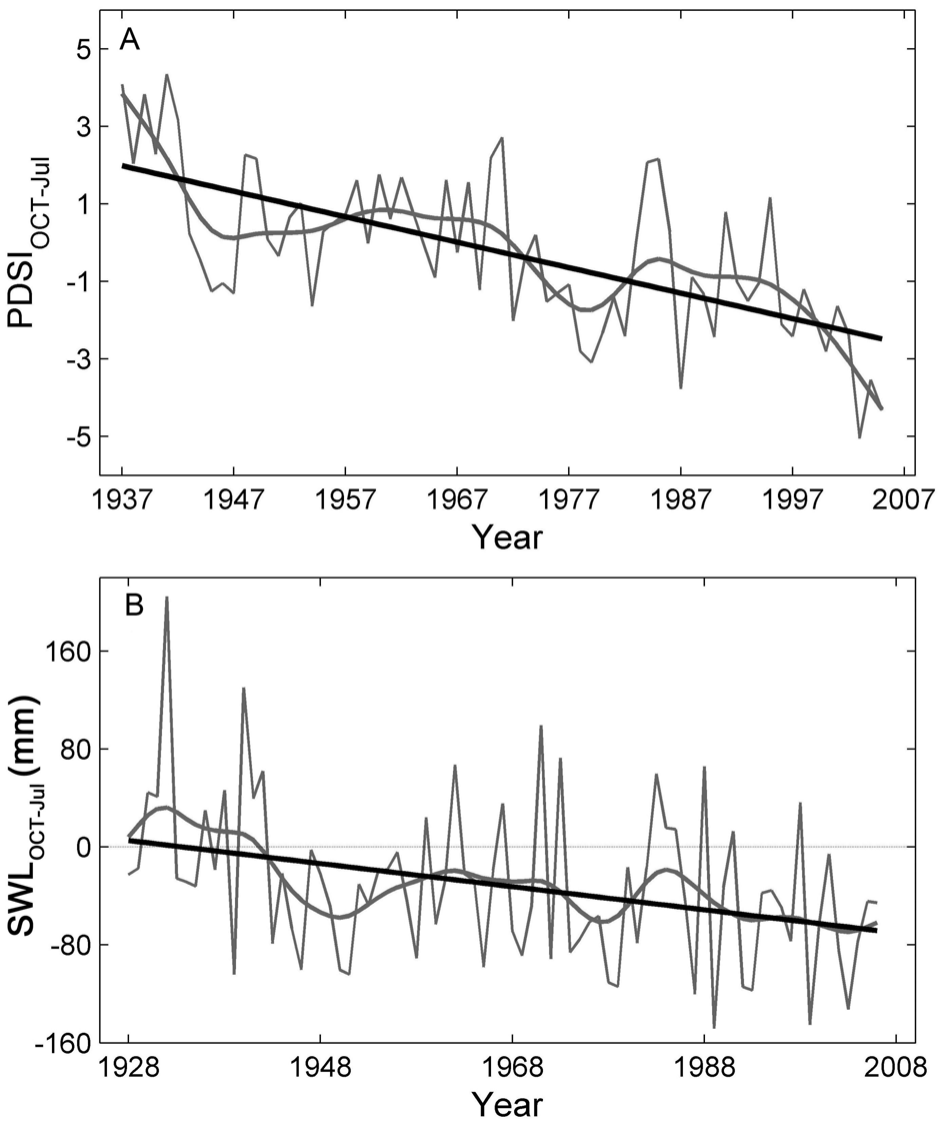
Changes of PDSI and soil water limitation in this region. Average PDSI values through October of prior year to July of current year during 1937–2005 (thin grey line) with a cubic smoothing spline (bold grey line) and a linear fit (black line, *y* = −0.066*x*+129.1, *p*<0.001) are shown in (A). Modeled regional soil water limitation through October of prior year to July of current year (SWL_OCT-Jul_) during 1928–2006 (light grey line) with the cubic smoothing spline (bold grey line) and a linear fit (black line, *y* = −0.94*x*+1823.7, *p*<0.01) are shown in (B).

The PDSI_OCT-Jul_ has been undergoing a dramatic decline since 1937, as detected by the Mann-Kendall test and highlighted by a smoothing cubic spline and a linear regression fit (Fig. 7A, *y* = −0.066*x*+129.1, *r* ^2^ = 0.42, *p*<0.05). The modeled regional soil water limitation through October of the previous year to July of the current year (SWL_OCT-Jul_) shows a marked increase since 1928, despite the high variability evident in the record (Fig. 7B). In general, the SWL_OCT-Jul_ for this region has increased at a rate of 9.4 mm per decade since 1928, as shown in the linear fit for SWL_OCT-Jul_ (Fig. 7B, *y* = −0.94*x*+1823.7, *r* ^2^ = 0.11, *p*<0.05).

## Discussion

### Drought Stress and Regional Tree Growth Decline

The increasing soil water limitation through October of the previous year to July of the current year, as indicated by the significant decreasing PDSI_OCT-Jul_ and increasing SWL_OCT-Jul_ (Figs. 7A, B), is tightly linked to the substantial decline in growth of hemi-boreal forests in southern Siberia, as demonstrated by the close relationships between regional tree growth and monthly PDSI and PDSI_OCT-Jul_ (Figs. 4C, D). The increasing soil water limitation has become more limiting to regional tree growth, which is indicated by the marked increase in the shared growth variance held by the chronologies of these three species (Fig. 4A), and by the matching trends in regional BAI and PDSI_OCT-Jul_ (Fig. 4D). These results suggest that drought stress limits the tree growth of hemi-boreal forests in our study region. Some previous studies in nearby regions have obtained similar results and corroborate this conclusion [9], [15], [16], [17], [18]. For instance, tree growth of *L. sibirica* in northern Mongolia [9] as well as that of *L. gmelinii* in central Siberia [18], was shown to decrease with increasing drought stress during the growing season. Notably, tree growth decline due to warming-induced drought stress is evident at mid- and even high- latitude areas of the Northern Hemisphere [6], [7], [8], [12], [19]. Taken together, these results support the hypothesis that drought-stress may accompany increased warming in the boreal forest [6], [19].

Why does temperature warming result in drought stress in hemi-boreal forests in southern Siberia and thus limit tree growth? Temperature increases are stronger in the pre- and early growing seasons in southern Siberia, without concurrent increases in precipitation. This leads to a marked increase in evaporation and, therefore, a dramatic decrease in soil water content (Fig. 7A) and an increase in soil water limitation (Fig. 7B). The dramatic increase in temperature during the pre- and early growing season may exacerbate the soil water limitation by degrading the permafrost [20], [21], [22] and reducing snow cover in our study region [23], whereas permafrost and snow melt could have a considerable effect on tree growth in this region [24], [25], [26]. Therefore, the pronounced warming, particular during the pre- and early growing seasons, appears to trigger a marked increase in soil water limitation in southern Siberia, which, in turn, has led to a recently marked decline in tree growth for these forests (Figs. 2A–F), notwithstanding that there are increasing trends in a proxy of soil water use efficiency (Fig. 6B, D, F). These results also highlight the important effect of environmental conditions before, and at the very beginning of, the growing season on tree growth and vegetation activity in hemi-boreal forests. Previous studies corroborate this conclusion and have shown that tree growth for *L. sibirica* in this region is mainly controlled by the precipitation received early in the growing season [27].

Clearly, we found no evidence for the widely-observed vegetation greening trends reported for the past two decades for southern Siberia [4], [5]. Instead we found evidence for a “browning” phenomenon, as indicated by the common decline in tree growth for these three dominant species over the last two decades (Figs. 2A–F). Remote sensing observations have revealed that the forested areas across northern circumpolar high latitude regions (above 50°N) have also suffered a gross “browning” trend in recent decades (i.e. during early 1980 s to early 2000 s) [28], [29], [30], particularly in the late summer, due to warming-induced drought [30].

The marked recent decline in tree growth for the three dominant species in hemi-boreal forests (Figs. 2A–F) due to warming-induced drought stress suggests that, under climate warming, the carbon uptake in this region may be stalled or even reversed, which would act as a positive feedback effect for climate warming [31]. If this limitation in growth due to drought stress is sustained, the future capacity of hemi-boreal forests to sequester carbon may be less than currently expected, although many other components of the carbon balance need further investigation. More importantly, increasing soil water limitation appears to have constrained forest regeneration [9], [17], [32] and increased the climate sensitivity of tree growth in this region (Figs. 6B–F). These results suggest that tree growth in this region is becoming more vulnerable to climate warming [9], [17], [32]. The present study, combined with previous results [9], [32], also suggests that the hemi-boreal forests will retreat if faced with more severe drought stress in the future.

Climate change will also indirectly alter forest dynamics through the effect on disturbance regimes [33], [34]. In boreal forests, the currently dominant disturbances are fire [35] and insects [36]. Although the recent common decline in tree growth for these three tree species cannot be linked to fire or insect disturbance (due to the lack of any evidence of fire, e.g. fire scars on trees or charcoals in the soil sediments), or to insect prevalence in our sampling sites, the incidence and severity of fires and insects is likely to increase with increased aridity [37], [38]. These disturbances, combined with warming-induced drought stress, may have a multiplicative effect on forest dynamics in hemi-boreal forests.

### Different Growth-climate Responses Among Species Linked to Stand Conditions

The growth response of different species to climate changes in the region differed, with the most obvious difference being between *P. sylvestris* and the two larch species. Comparisons of the growth-climate relationships found for individual species show that *P. sylvestris* growth tends to be more limited by soil water limitation than growth for the larch species (Figs. 5A–C). Several factors, such as differences in the fire and insect regime, tree density, stand-level water conditions and drought resistance capacity, can contribute to differences in tree growth patterns and to growth-climate relationships. As mentioned above, we did not find any evidence of fire or insect disturbance at any of our sampling sites, therefore the different tree growth pattern and growth-climate relationship cannot be linked to the difference between the fire and insect regimes for the pine and larch forests. In addition, the tree density of the pine forests (canopy cover of 25%∼35%) is much lower than that of the larch forests (canopy cover of 50%∼70%), indicating that the more severe drought stress (Fig. 5A) in pine forests can not be attributed to higher tree competition. Instead, these differences may be largely due to differences in stand-level water conditions and the drought resistance capacity inherent in each species.

*P. sylvestris* growing in the forest–steppe ecotones exhibits conservative water consumption with a sensitive stomatal regulation and relatively constant shoot water potentials under dry conditions [39]. It has a much lower transpiration rate (0.06–0.18 g.g^−1^ h^−1^) than the two larch species (*Larix sibirica* 0.06–0.54 g.g^−1^ h^−1^ and *Larix gmelinii* 0.10–1.08 g.g^−1^ h^−1^) [40]. These results suggest that *P. sylvestris* has greater drought resistance [39], [40].

The *P. sylvestris* forests are generally located on the north-facing slopes of mountains in the forest-steppe ecotones, where permafrost islands seldom melt within the valley bottoms, which are covered by peaty soils. Therefore, *P. sylvestris* trees at the sampling sites are not influenced by permafrost but are only influenced by the seasonal frozen soil [41]. In winter a seasonal soil freezing up to a depth of 2–2.5 m occurs [41]. In comparison, the larch forests are generally located in a zone with sporadic permafrost (*L. sibirica*) or discontinuous permafrost (*L. gmelinii*), with a permafrost depth of 50–80 m and 120–130 m, and mean annual temperature of −0.2 to −1°C and −1.2 to −1.7°C respectively [41]. During summer, the depth of the active layer of permafrost is 1.5–2.5 m [41], [42]. A previous study has proven that permafrost can be a direct source of water for plants under severe drought stress, and can retain surplus water in the soil until the next summer [24]. Therefore, the larch forests could benefit from the permafrost, especially during the mid to late growing season [24]. Although the pine forests could also benefit from seasonal frozen soil, there are two reasons that they may face a more severe drought. Firstly, the pine forests are located in a drier region, which suffers from a more rapid early spring warming (unpublished data from our group), leading to a much earlier melting of the seasonal frozen soil, and thus to drier soil conditions due to evaporation and possible runoff in the well-drained soil. Secondly, the organic layer depth is much shallower (0∼2 cm) in the pine forest areas than in the larch forests areas (5∼10 cm). This difference makes the melting of the seasonal frozen soil in pine forests easier and causes it to occur earlier than the analogous melting of permafrost in larch forests, because the net effect of the organic layer is to lower the soil temperature and decrease the seasonal thaw depth [22]. Taken together, differences in soil water conditions are probably the main explanation for the different tree growth responses to the changing climate between the Scots pine and larch.

These results can give us some insight into vegetation dynamics [43] and can be useful for the devising of adaptive forest management strategies for this region. With increasing drought stress (Figs. 7A, B) and climate sensitivity (Figs. 6A, B), *P. sylvestris*, which dominated the forest-steppe ecotone, is currently unable to encroach on the steppe in this region. Predicted stronger climate warming trends [11] could result in a retreat of the pine forest in the forest-steppe ecotones due to more severe warming-induced drought stress. The larch forests in this region will become more vulnerable when facing increased climate warming and will suffer a long-lasting decline in growth. In addition, if a retreat of the permafrost were to accompany the warming climate, a reduction in these forests could follow [44]. However, redistribution of forest zones and their dominant climates will require long periods of adjustment for the amount of change being predicted [44]. Given the potential risks of climate-induced forest decline, increased attention should be paid to forest management for this region. For the much denser larch forests, a light thinning management, which moderates competition for water between trees, could be an alternative option for enhancing forest resistance and resilience to increasing climate stress [45]. For the pine forests (with canopy cover of 25∼35%), we advise selecting more drought-resistant genotypes to adapt the drier soil conditions that *Pinus sylvestris* is facing [46].

## Materials and Methods

### Study Area and Sample Collection

The sample area was a northeast-southwest transect in the Trans-Baikal region, which is located at the southern limit of the boreal forest, also called hemi-boreal forest (Fig. 1A). Three widely distributed coniferous species, *Larix sibirica, L. gmelinii* and *Pinus sylvestris,* which dominate the Trans-Baikal coniferous forests, East Siberian taiga and Selenge-Orkhon forest steppe, respectively, are found in this region. Forests in this area are generally fragmented and distributed on the north-facing slopes of mountains. Pine forests are generally located in much drier regions than larch forests. In addition, the canopy cover of pine forests (25%∼35%) is much lower than that of larch forests (50%∼70%). The soil is coarse-textured and poor in nutrients, with a total nitrogen content ranging from 0.5 to 2.7 g/kg and a total carbon content ranging from 10 to 38 g/kg.

The climate in this region is continental. Mean annual precipitation is about 446 mm. July (mean temperature of 16.7°C) and January (mean temperature of −24.8°C) are the warmest and coldest months respectively (Fig. 1B). The growing season is approximately May-September, during which about 79% of the mean annual precipitation is received. In addition, there are discontinuous areas of permafrost in our study region, with the mean annual ground temperature ranging from 0 to −2°C [22].

Tree-ring samples from three different tree species were collected from 8 representative stands of 25 × 25 m^2^ (Fig. 1A). In these selected stands human and animal disturbance (e.g. grazing, logging) is minimal (few stumps and no feces evident). In addition, we found no evidence of disturbance by insects or fire (no fire scars evident on trees or charcoals in the soil profiles). However, we cannot be certain of the disturbance regime of these forests before tree establishment. The primary objective of this study is to evaluate patterns of tree growth in this region, and to assess their relationship to climate change. In each stand, we sampled all trees with a d.b.h (diameter at breast height) greater than 10 cm unselectively using an increment borer at a height of 1.3 m. In general, 30–80 individuals were sampled from each stand. Two cores were taken from each tree. The geographical features of the sampling sites are shown in Table 1. Notably, no specific permits were required for the presented field studies, and the field studies did not involve endangered or protected species.

**Table 1:** Geographic features and chronology statistics for our study sites.

Sites						Chronology†				
Site ID	Latitude (N)	Longitude (E)	Altitude (m)	Slope	Slope Degree	No.Trees	No.Radii	*ms_x_*	*R*	Reliable*
										Time Span
*L. sibirica*						178	306	0.428	0.675	1928–2006
Plot A	50.63°	104.35°	858	NE 25°	26°	84	168	0.42	0.84	1923–2006
Plot B	50.57°	104.67°	1035	NE 0°	15°	45	87	0.43	0.573	1924–2006
Plot C	50.58°	104.88°	876	NE 0°	20°	49	69	0.452	0.775	1928–2006
*P. sylvestris*						87	165	0.432	0.515	1910–2006
Plot D	50.87°	105.98°	920	NE 50°	15°	35	55	0.473	0.871	1900–2006
Plot E	51.16°	106.54°	834	NE 20°	15°	58	103	0.36	0.553	1928–2006
Plot F	51.70°	107.16°	751	NE 30°	20°	27	45	0.45	0.575	1927–2006
*L. gmelinii*						120	200	0.423	0.523	1928–2006
Plot G	52.49°	111.58°	1038	NE260°	10°	54	92	0.372	0.504	1910–2006
Plot H	52.89°	112.10°	991	NE17°	3°	44	81	0.311	0.573	1892–2006

Note:

† Standard ring-width chronologies were developed for eight stands (A-H) using conservative detrending methods based primarily on the negative exponential function or linear regression with any slope. Species-specific standard chronologies were developed for *L. sibirica*, *L. gmelinii* and *P. sylvestris*, respectively.

* We determined the reliable time span for built chronologies according to the criteria of EPS (Expressed Population Signal) >0.85.

### Chronology Construction and Tree Growth Evaluation

All samples were cross-dated, measured (with precision to the nearest 0.01 mm, using the LINTAB system) and processed using standard dendrochronological techniques [47], [48]. Using the program ARSTAN, we applied conservative detrending methods, based primarily on the negative exponential function or on a fitted linear regression with any slope for each raw measurement series, to remove non-climatic and tree-age-related growth trends [49]. Tree ring indices (TRI) were obtained by dividing the observed ring-width value by the predicted ring-width value. For each stand, the TRIs were averaged by year using a bi-weighted robust mean to construct a standard chronology. The Expressed Population Signal (EPS), with a threshold set at 0.85, was used to determine the most reliable time span for the chronologies [50], [51]. We then constructed the standard ring-width chronologies for the three different tree species by averaging the standard chronologies for the relevant sites to determine the tree growth trend and annual variability. Descriptive statistics are presented to allow comparisons of the sites with other dendroclimatic data sets (Table 1).

Given the bias intrinsic to investigating tree growth trends on the basis of changing tree ring indices alone [52], we compared and combined the basal area increment (BAI) and tree ring indices to evaluate long-term changes in growth [52], [53], [54]. BAI is far less dependent on changes in tree age/size and is a good indicator of forest growth and productivity [53], [54].

Stem BAI was computed using the cross-dated ring width series. Past annual BAI was estimated by subtracting twice the annual ring width from the annual outside bark diameter [12]. Raw BAI chronologies for the three species were built as the average by year of individual-tree BAI series to establish the long-term growth trends. Smoothing cubic spline functions were fitted to the BAI and ring-width chronologies for the three species using MATLAB, in order to highlight the long-term growth trends.

The high quality GIMMS (Global Inventory Modeling and Mapping Studies) dataset of the normalized difference vegetation index (NDVI) [55], [56] was compared to the species BAI series. Since NDVI has been demonstrated to be a high quality indicator of large-scale trends in vegetation activity, this comparison allowed us to assess whether the sample data obtained here are representative of larger scale tree growth patterns [57].

### Climate Data

Observed climate data from the nearest stations (Fig. 1A) were obtained from the National Climatic Data Center (NCDC, http://www.ncdc.noaa.gov/). However, most available climate records in this region are only available after 1947, and are surprisingly discontinuous, and so cannot be straightforwardly matched for the period for our growth data (1928–2006). To gain a more thorough understanding of the relationship between climate and tree growth, we introduced a monthly gridded dataset from the Climate Research Unit (CRU TS 3.0, 1901–2006), with a regular latitude-longitude resolution of 0.5°×0.5° (CRU, http://www.cru.uea.ac.uk/) [58]. Regional monthly temperature and precipitation data derived from CRU TS 3.0 appear highly similar to the instrumental records from meteorological stations in this region for 1950–2006, with correlation coefficients for monthly temperature ranging from 0.89 (*p*<0.000001) in September to 0.98 (*p*<0.000001) in March, and for monthly precipitation ranging from 0.68 (*p*<0.001) in January to 0.92 (*p*<0.000001) in August. Based on a careful comparative analysis, monthly temperature and precipitation data from 1928 to 2006 were derived from the nearest grid cells of CRU TS 3.0 (Fig. 1A).

Monthly Palmer Drought Severity Indices (PDSI) for 6 grids (Fig. 1A) from 1937 to 2005 were derived from a global PDSI dataset with a spatial resolution of 2.5° × 2.5° [59]. The PDSI is a good indicator of long-term regional soil water conditions because it incorporates the coupling effects of precipitation, temperature, and potential evapotranspiration [59], [60], [61], which are generally considered to be sensitive, low-noise indicators of large-scale interannual variations in soil water content in central Asia [59], [60]. We also compared and validated the PDSI using the available gravimetric measurements of soil water contents in our study region.

We obtained monthly soil moisture observations for the uppermost 1 m layer of soil from the RUSWET-GRASS dataset [15], [62] (http://www.ipf.tuwien.ac.at/insitu/). This dataset contains gravimetric measurements of natural grass available soil moisture from 1978 to 1985, taken at 130 meteorological stations in the former Soviet Union. Observations were made with a temporal resolution of about 10 days during the warm season, and once a month during winter. Four points in each flat observational plot (about 0.1 ha for each plot) are used for each data point, and the results averaged to give the data value. In this study, we used soil moisture observations from 8 sites which are close to our sampling plots and to the selected PDSI grids (Fig. 1A). In most cases, only warm season soil moisture data were available for these sites. Therefore, we evaluated and calculated the growing season (May-September) average soil moisture for each site and validated the relevant PDSI data. In the growing season, the average PDSI values correlated significantly and positively with the observed soil water content for the period 1978–1985 (Fig. *S1*, *r* ^2^ = 0.34, *p*<0.001), suggesting that the PDSI values can be used to indicate changes to soil water content in our study region.

The monthly soil water limitation (SWL) for this region for each biological year (running from October of the previous year to September of the current year) in the period 1928–2006 was estimated based on a simple water balance model:
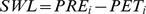
where *PRE_i_* is the total precipitation for month *i* and *PET_i_* is the potential evapotranspiration for month *i*, which is calculated using the Thornthwaite formula [63]. Specifically, there is surplus water when the monthly potential evapotranspiration is less than the total precipitation, otherwise there is a water shortage. The dynamic water balance was then calculated month by month and the total water limitation was estimated over months and seasons by summing the monthly SWL. Trends in monthly and seasonal climate series (i.e. temperature, precipitation, PDSI and SWL) were first identified by the Mann-Kendall non-parametric test [64], [65]. Where a significant trend in a climate series was detected following the Mann-Kendall test, a linear regression was fitted and the slope estimated.

### Relationships between Regional and Species Tree-growth and Climate Variables

A principal component analysis based on the correlation matrix was carried out for the 8 standard chronologies for the period 1928–2002, in order to evaluate regionally shared growth variability, which was assumed to correspond to the value of the first principal component (PC1) [66]. The broken stick test was performed to determine the significance of each of the principal components [67]. Temporal changes in shared growth variability were investigated using subintervals of 20 years with a 5-year lag. The subinterval of 20 years is required because the number of observations (years) must be greater than the number of variables (chronologies) in order to meet the requirements of the statistical analyses. The variance explained by PC1 was considered to be an indicator of the similarity between the 8 chronologies. Correlation analyses between the regional chronology (PC1) and species chronologies and climate series (monthly temperature, precipitation and PDSI) were conducted using the program DENDROCLIM2002 to identify the climate driver for regional and species tree-growth [68].

### Climate Sensitivity of Tree-growth

In this study, we assessed tree growth sensitivity to climate using both annual sensitivity (*S_x_*) and the proxy of soil water use efficiency (P_SWUE_). The annual sensitivity (*S_x_*) is the relative difference between one ring-width index and the next, and was calculated using the formula:
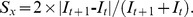
where *I_t_* is the ring-width index value for the year *t* [47]. This index is frequently used in dendroclimatology to show tree-growth sensitivity to climate [47]. Higher values for this index are indicative of greater interannual changes in ring-width, which imply a biased biological tree-growth rate. The proxy of soil water use efficiency was calculated from the following formula:



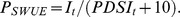
where *I_t_* is the ring-width index value for year *t*, and *PDSI_t_* is the biological-year annual average value for year *t*. We introduced a constant of 10 into the formula in order to offset negative PDSI values, as PDSI is a standard measure of surface moisture conditions, theoretically ranging from about −10 (dry) to 10 (wet) [59].

There are close positive relationships between both ring width index and NDVI [57], [69], and NDVI and woody biomass [1], [70] in boreal forests. Changes in ring width index therefore suggest variations in woody biomass in boreal forests, and changes in PDSI can indicate variations in the soil water content for our study region, as illustrated above (Fig. *S1*). P_SWUE_ is a reasonable proxy for soil water use efficiency for tree growth. We further validated the constructed P_SWUE_ by comparing it against the ratio of BAI (a measure closely related to forest production) to soil water content for growing seasons over the period 1978–1985. The ratio of BAI to soil water content is a more physically grounded index that generally gives the true soil water use efficiency. For all the three species, there are close positive relationships between P_SWUE_ and the ratio of BAI to soil water content (Fig. *S2*, *p*<0.05 for all the three species). This confirms that the introduced measure of P_SWUE_ is a suitable and appropriate proxy for soil water use efficiency.

### Conclusions

Our study found that hemi-boreal forests have suffered a marked decline in growth over the last two decades. Increasing soil water limitation during pre- and early growing seasons triggered by climate warming, contributed most significantly to this decline in growth. This rapid growth decline, and increased climate sensitivity, mean that these hemi-boreal forests may become more vulnerable if faced with greater drought stress in the future, as predicted by climate models [11]. In addition, different tree species differ in their growth patterns and responses to the changing climate. *P. sylvestris* suffers a more severe drought limitation than the larch species (*L. sibirica* and *L. gmelinii*), which can mostly be attributed to differences in soil water conditions. These findings provide valuable insights into the regional carbon cycle and vegetation dynamics, and could be useful for devising adaptive forest management strategies. The present study, combined with previous results [9], [32], [44], suggests that the hemi-boreal forests will retreat if faced with more severe drought stress and with potentially increasing levels of disturbance (e.g. fire and insect). Given the potential risks of climate-induced forest decline, increased attention should be paid to the management of adaptation options for enhancing forest resistance and resilience to projected climate stress. At stand level, we suggest reducing tree densities by thinning, thus moderating competition for water for the larch forests, and selecting more drought-resistant genotypes for pine forests. At a regional scale, we need to identify the areas where tree-growth decline is most likely to occur by monitoring climate trends, changes in permafrost and in the disturbance regime, and tree-growth patterns.

## Supporting Information

Figure S1 **Comparison between measured soil water contents of growing season for the 8 sites and the average PDSI values of growing season for the relevant grids during 1978–1985.** Line in figure indicates the linear fit of this relationship (*y* = 0.91*x*+11.77, *r* ^2^ = 0.34, *p*<0.001).
(TIF)

Figure S2 **Comparisons between the constructed P_SWUE_ and the ratio of basal area increments (BAI) to soil water contents for different species during 1978–1985.** Lines in the figure indicate the linear fits of the relationships for *Pinus sylvestris* (grey line, *y = *0.2*x+*0.066, *r* ^2^ = 0.58, *p*<0.05), *Larix sibirica* (black line, *y = *0.29*x*+0.018, *r* ^2^ = 0.60, *p*<0.05), and *Larix gmelinii* (light grey line, *y* = 0.18*x+*0.025, *r* ^2^ = 0.53, *p*<0.05).
(TIF)

Figure S3 **Scatter plots of principal component analysis (PCA) loadings of the 8 chronologies for the period 1928–2006.** *P. sylvestris*, *L. sibirica* and *L. gmelinii* chronologies are marked as squares, triangles, and circles, respectively.
(TIF)
