# Supplementary figures and images for: Growth Decline Linked to Warming-Induced Water Limitation in Hemi-Boreal Forests
Source: PLoS One. 2012 Aug 15;7(8):e42619. doi: 10.1371/journal.pone.0042619 (PMC3419722; doi:10.1371/journal.pone.0042619)

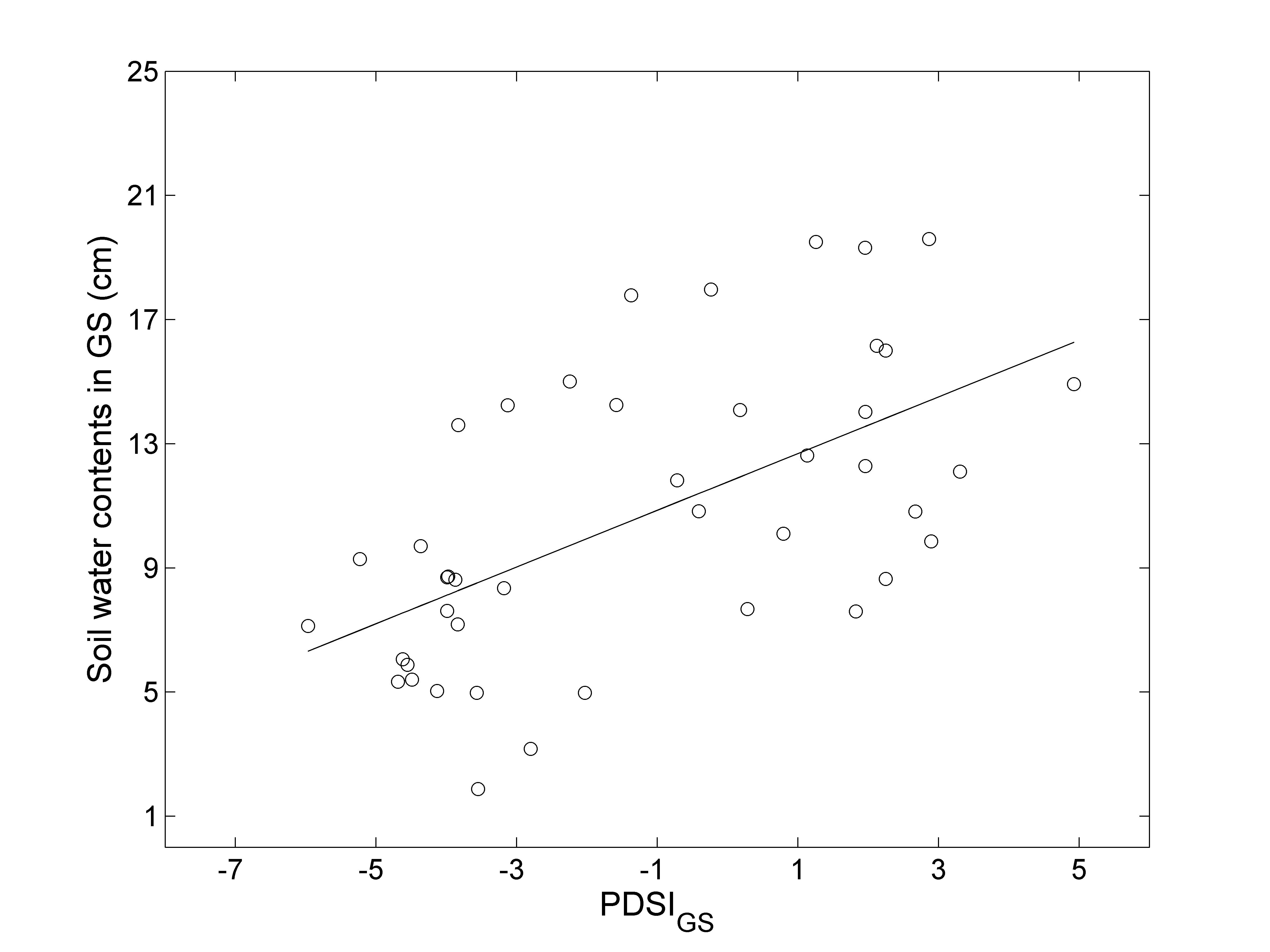

Supplement: Figure S1 — Comparison between measured soil water contents of growing season for the 8 sites and the average PDSI values of growing season for the relevant grids during 1978–1985. Line in figure indicates the linear fit of this relationship (y = 0.91x+11.77, r 2 = 0.34, p<0.001). (TIF) [file pone.0042619.s001.tif]

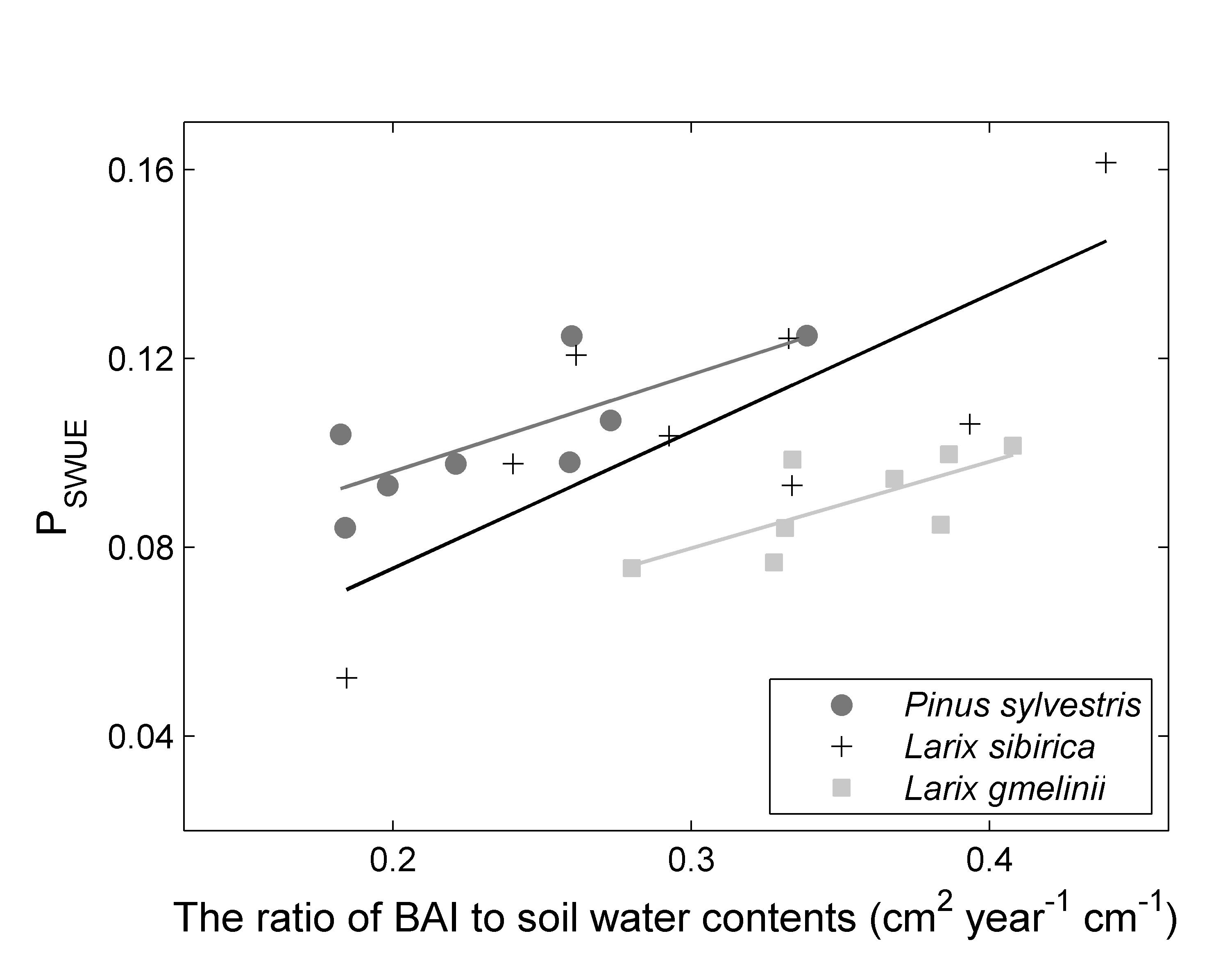

Supplement: Figure S2 — Comparisons between the constructed PSWUE and the ratio of basal area increments (BAI) to soil water contents for different species during 1978–1985. Lines in the figure indicate the linear fits of the relationships for Pinus sylvestris (grey line, y = 0.2x+0.066, r 2 = 0.58, p<0.05), Larix sibirica (black line, y = 0.29x+0.018, r 2 = 0.60, p<0.05), and Larix gmelinii (light grey line, y = 0.18x+0.025, r 2 = 0.53, p<0.05). (TIF) [file pone.0042619.s002.tif]

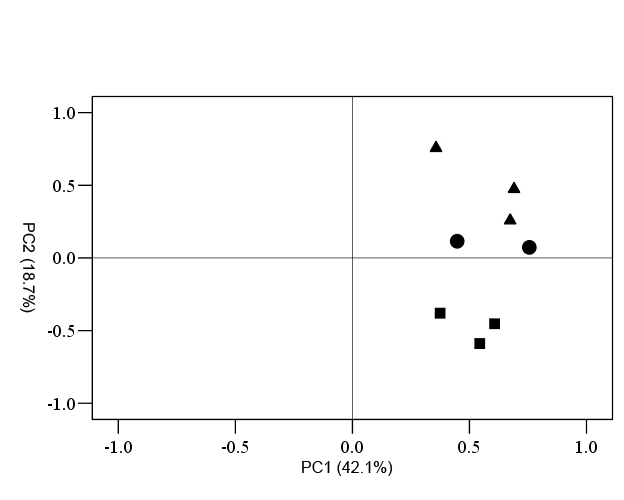

Supplement: Figure S3 — Scatter plots of principal component analysis (PCA) loadings of the 8 chronologies for the period 1928–2006. P. sylvestris, L. sibirica and L. gmelinii chronologies are marked as squares, triangles, and circles, respectively. (TIF) [file pone.0042619.s003.tif]
